# The effects of topical dorzolamide 2% and brinzolamide 1%, either alone or combined with timolol 0.5%, on intraocular pressure, pupil diameter, and heart rate in healthy cats

**DOI:** 10.1111/vop.12679

**Published:** 2019-05-17

**Authors:** Inge J. M. Slenter, Sylvia C. Djajadiningrat‐Laanen, Daphne J. Elders, Reinoud de Gee, Linda E. Koele, Loes W. Vermeer, Michael H. Boevé

**Affiliations:** ^1^ Ophthalmology Section, Department of Clinical Sciences of Companion Animals Utrecht University Utrecht The Netherlands; ^2^ Faculty of Veterinary Medicine Utrecht University Utrecht The Netherlands

**Keywords:** β‐blocker, carbonic anhydrase inhibitor, feline, glaucoma

## Abstract

**Objective:**

To investigate the effects of topical dorzolamide 2% q8h and brinzolamide 1% q8h, administered either alone (A and B, respectively) or in combination with topical timolol 0.5% q12h (C and D, respectively), on the circadian pattern of intraocular pressure (IOP), the pupil size, and heart rate in healthy cats.

**Methods:**

In this prospective, randomized, double‐blinded study, 10 healthy, adult cats were randomly assigned to one of four groups and the eye to be medicated was randomly assigned. IOP, pupil diameter, and heart rate were measured at 3‐hour intervals. A 5 days’ adjustment period was followed by a 5 days’ placebo (baseline) period. Then, all groups of cats received all four treatments (A‐D) according to a Latin square‐based rotating schedule. Five days’ medication periods were alternated with 3 days’ washout periods.

**Results:**

Mean baseline IOP was 13.6 ± 2.7 mm Hg. All treatments resulted in a statistically significant decrease in mean IOP in the treated eye: A: −2.33 mm Hg (95% CI: −2.71, −1.94), B: −1.91 mm Hg (95% CI: −2.30, −1.53), C: −2.36 mm Hg (95% CI: −2.74, −1.97), and D: −2.37 mm Hg (95% CI: −2.76, −1.98) and the nontreated eye: A: −0.19 mm Hg (95% CI: −0.28, −0.11), B: −0.18 mm Hg (95% CI: −0.27, −0.10), C −0.31 mm Hg (95% CI: −0.40, −0.23), and D: −0.24 mm Hg (95% CI: −0.32, −0.15). Timolol resulted in an additional, significant decrease in IOP of 4% and 5%, respectively, compared to A and B, and in mild bradycardia and miosis.

**Conclusions:**

Topical administration of dorzolamide 2% and brinzolamide 1% q8h significantly decreased IOP in healthy cats. Supplemental timolol 0.5% eye drops q12h resulted in an additional, statistically significant reduction of IOP.

## 1 INTRODUCTION

Glaucoma is a painful and blinding disease in cats characterized by an increase in intraocular pressure (IOP) and neuroretinal damage.^1^, ^2^, ^3^ Chronic uveitis and intraocular neoplasia appear to be the most common causes of feline glaucoma, whereas primary glaucoma in cats is rare.^4^, ^5^, ^6^, ^7^ Medical treatment of glaucoma secondary to sequelae of chronic uveitis is challenging. Topically administered carbonic anhydrase inhibitors and beta‐adrenergic blockers are currently among the most commonly used agents.^1^, ^3^ These agents lower aqueous humor production in the ciliary body by, respectively, inhibiting active, carbonic anhydrase‐mediated aqueous humor formation^8^ and—presumably—altering adrenergic neuronal control of aqueous humor production.^9^

Efficacy studies have been performed, but results are conflicting, and may be difficult to compare due to small sample sizes and differences in study design. Dorzolamide 2%, administered q8h, significantly decreased IOP in both healthy cats and cats with congenital, primary glaucoma.^10^, ^11^, ^12^ By contrast, q12h or q8h administration of brinzolamide 1% did not reduce IOP in healthy cats, but q8h administration significantly decreased IOP and diurnal IOP fluctuation in cats with congenital, primary glaucoma.^13^, ^14^ A single drop of a 0.5% timolol solution resulted in a significant decrease in IOP in normal cats^15^; however, when applied as a gel‐forming solution q24h for 8 days, timolol had an inconsistent effect on IOP in both normal and glaucomatous cats.^16^ Considering the different mechanisms of action, the combined administration of a carbonic anhydrase inhibitor and a beta‐adrenergic blocker would expectedly result in an additive IOP‐lowering effect. However, in healthy cats, the combined topical administration of dorzolamide 2% q8h and timolol 0.5% q12h for 8 days did not result in a significantly greater decrease in IOP than the q8h administration of dorzolamide 2% alone.^11^ As far as the authors know, the effects of combined topical administration of brinzolamide 1% and timolol 0.5% have not been investigated in cats.

The IOP in healthy cats demonstrates a circadian fluctuation in the order of 4 mm Hg,^17^ with peaks reported in the morning,^11^, ^13^ during the evening,^14^, ^15^, ^18^ or at night.^12^, ^17^ IOP fluctuations in glaucomatous cats reportedly are two to eight times higher than in healthy cats.^12^ It is conceivable that levelling off the circadian IOP peaks in glaucomatous individuals may aid in preserving retinal ganglion cell and optic nerve function. However, there is a paucity of studies on the effect of IOP‐lowering drugs on the circadian IOP in cats.^12^, ^14^

The aim of our study was, therefore, to expand the current knowledge on treatment options for glaucoma in cats by investigating the effects of dorzolamide 2% and brinzolamide 1%, administered either alone or in combination with timolol 0.5% eye drops on the circadian rhythm of the IOP in normal cats.

## 2 MATERIALS AND METHODS

### 2.1 Animals

The study population comprised ten 12‐ to 19‐month‐old domestic shorthaired cats kept for teaching purposes by the Department of Clinical Sciences of Companion Animals of the faculty of Veterinary Medicine of Utrecht University. The five castrated male cats and five neutered female cats were housed in two sex‐based groups in two separate large kennels with both an indoor and outdoor facility. The kennels and the examination room were illuminated by a combination of natural and artificial light. During the study period, daytime length, counted from the onset of civil dawn to the end of civil dusk (ie, from the moment when the geometric center of the sun is six degrees below the horizon in the morning to the moment when the center of the sun reaches six degrees below the horizon in the evening), varied between 10.5 and 13 hours. Artificial lights were switched on at 8.00, which was one hour after civil dawn, and were turned off at 19.00, which was one hour before to 1.5 hours after civil dusk. Artificial lights were briefly switched on during transportation of the cats to and from the examination room and during examination.

This study adhered to the guidelines laid down by our institution's animal care and use committees: the Animal Ethics Committee (DEC), the Animal Welfare Body (IvD), and the Central Authority for Scientific Procedures on Animals (CCD).

### 2.2 Study design

This was a prospective, randomized, double‐blinded study. The 10 cats were randomly divided into four groups using a Random Team Generator (https://www.jamestease.co.uk/team-generator/). The eye to be medicated was randomly assigned using the flip‐a‐coin method.

The study period comprised 42 days and was divided into a 5 days’ adjustment period, a 5 days’ placebo period during which baseline values were obtained, and four 5 days’ treatment periods. There was one day of rest following the adjustment period, there were two days of rest following the placebo period, and three washout days between every treatment period.

### 2.3 Ophthalmic examination

Prior to the study and after each medication period, all cats were examined by a board‐certified veterinary ophthalmologist (MB, SD). Ophthalmic examination included measurement of aqueous tear production (Schirmer Tear Test, MSD Animal Health BV,), fluorescein staining (BIO FLUORO, Fluorescein Sodium Ophthalmic Strip USP, BIOTECH, Gujarat, India), rebound tonometry (TonoVet, ICare), slit‐lamp biomicroscopy (SL‐17 portable slit lamp, Kowa Company Ltd.), and indirect ophthalmoscopy (Video Omega®2C, Heine Optotechnik GmbH & Co. KG).

### 2.4 Parameters measured

Intraocular pressure, horizontal pupil diameter, light intensity, and heart rate were measured at 3‐hour intervals at 9.00, 12.00, 15.00, 18.00, 21.00, 0.00, 3.00, and 6.00. Measurements were performed by two teams of two examiners each (DE, LK, LV, RG) and occasionally by two supplemental examiners (IS, SD). Measurements were performed during the 5 days’ adjustment, placebo, and medication periods and on the last day of the 3 days’ washout periods (zero measurement). Cats were examined in a fixed order, as were the eyes (right eyes first).

### 2.5 Drug administration

Eye drops were administered at 7.30, 7.45, 15.30, 19.45, and 23.30.

During the placebo period, the eye to be medicated received a topical artificial tear solution (Lacriforte®, AST Farma BV) 5 times daily.

In the subsequent medication periods, all groups of cats received four treatments according to a Latin square‐based rotating schedule. The original labels of all eye drops were removed and replaced by blank labels mentioning only the name of the cat and the eye to be treated. Treatment protocols included topical administration of one drop of (A) dorzolamide hydrochloride 2% (Dorzolamide 20 mg/mL, Centrafarm BV) q8h and artificial tear solution q12h, (B) brinzolamide hydrochloride 1% (Azopt®, Alcon) q8h and artificial tear solution q12h, (C) dorzolamide hydrochloride 2% q8h and timolol maleate 0.5% (Timolol Sandoz® 5 mg/mL, Sandoz) q12h, and (D) brinzolamide hydrochloride 1% q8h and timolol maleate 0.5% q12h (D). The contralateral eye received a topical artificial tear solution (Lacriforte®, AST Farma BV) at all five time points.

Immediately following the application of an eye drop, the cats were observed for signs of ocular discomfort. Signs were recorded using a scale of – to ++++ (no blinking or a single blink = ‐; 5 seconds of blinking = +; 30 seconds of blinking/squeezing = ++; >30 seconds of blinking/squeezing = +++; >30 seconds of squeezing and/or fighting = ++++).

### 2.6 Intraocular pressure

For IOP measurements, the cats were gently manually restrained in a normal upright position, the chin resting on the handler's index fingers, and the eyes gazing forward. Care was taken not to exert any pressure on the jugular veins or on the globe during manipulation of the eyelids, in order to avoid an artificial increase in IOP.^19^ A rebound tonometer (TonoVet, ICare®) was used according to the manufacturer's recommendations, with the exception of frequency of probe replacement. The “d” or dog setting was used, and only results with a deviation ≤1.0 mm Hg were included. For each cat, three consecutive average readings of the right eye (OD) were followed by three consecutive average readings of the left eye (OS). During the adjustment period, it was noted that the IOP in OD tended to be consistently higher than the IOP in OS. Therefore, the protocol was altered for the remainder of the study, and the first set of three consecutive measurements (IOP1) for both eyes (OU) was followed by a second set of three consecutive measurements OU (IOP2), which were obtained in the same order (OD first) as IOP1 measurements. After three consecutive double sets of measurements, the TonoVet probe was replaced.

### 2.7 Pupil diameter

Horizontal pupil diameter was measured at the vertical center of the pupil with a digital caliper (kwb Germany GmbH) positioned at a maximum of 2 mm anterior to the cornea.

### 2.8 Ambient light intensity

The light intensity at eye level was evaluated with the use of a lux meter (MASTECH® MS6610 digital lux meter, Mastech Digital).

### 2.9 Heart rate

Heart rate was obtained by counting the heart beats for 15 seconds per thoracic auscultation (3M™ Littmann® stethoscope, model Classic II SE, Medisafe LLP) and multiplying by 4 to obtain the heart rate in beats per minute (bpm).

### 2.10 Statistical analysis

Data were analyzed in R (version 3.2.2) using the lmer function from the *lme4* package (R Development Core Team [2008]).^20^, ^21^ Linear mixed‐effects regression models were used to analyze the effect of the various treatment protocols on IOP, heart rate, and horizontal pupil diameter. For all treatment periods, the data of the treated and nontreated eye of days 3‐5 were compared to days 3‐5 of the placebo period. The IOP used for analysis was the mean of the second set of three consecutive tonometric readings for that eye (IOP2). Fixed effects included day, treatment, time, and treated eye. Day 3 and time period 1 (9.00) were set as intercept. Random effects: animal and random slope: time with animal. Log transformations were used to compare the horizontal pupil diameter of the treated and nontreated eye and the heart rate from day 3 to 5. To compare the different treatment protocols with each other (placebo period excluded), log transformations were used to evaluate the IOP and horizontal pupil diameter of days 1‐5. In the regression models, the pupil diameter was corrected for the influence of light intensity.

Paired t tests were used to compare the IOP on days 1‐2 with the IOP on days 3‐5 in the treated and nontreated eyes, in both the adjustment period and the placebo period. As IOP2 readings were not performed during the adjustment period, mean data from the first set of three consecutive tonometric readings (IOP1) were used for this analysis. Paired t tests were also used to analyze differences between IOP1 and IOP2 for the treated and nontreated eyes during the placebo and all treatment periods. One‐way ANOVA was performed to analyze the maximal IOP‐lowering effect of the treated eye only, on the first day of treatment for all treatment protocols.

## 3 RESULTS

### 3.1 Animals

Before and during the study, ocular health was confirmed in all cats. The original intention was to include 12 cats in our study; however, during the placebo period, two cats (one male, one female) turned out not to be compliant with the administration of a topical solution and they were therefore excluded from the study.

In one of the, apparently healthy, male cats, a heart murmur was noticed during thoracic auscultation. Ultrasonographic examination of the heart by an ECVIM‐CA (cardiology) Diplomate revealed concentric hypertrophy of the left ventricle, systolic anterior movement (SAM) of the mitral valve and an enlarged left ventricle, matching with either hypertrophic cardiomyopathy or mitral valve dysplasia. There were otherwise no clinical abnormalities, and the cardiologist deemed the cat fit for participation in the study without systemic medication.

### 3.2 Intraocular pressure

In the eye selected for medication, mean days 1‐5 IOP was 16.0 ± 3.7 mm Hg in the adjustment period and 14.9 ± 3.7 mm Hg in the placebo period. During both the adjustment period and the placebo period, mean IOP1 was significantly higher on days 1‐2 than on days 3‐5 (*P* < 0.001), with a mean difference of 2.0 mm Hg in the adjustment period and a mean difference of 1.5 mm Hg in the placebo period.

Mean days 1‐5 IOP1 was significantly higher in comparison with mean days 1‐5 IOP2 in the placebo group and all treatment groups (*P* < 0.001).

**Figure 1:**
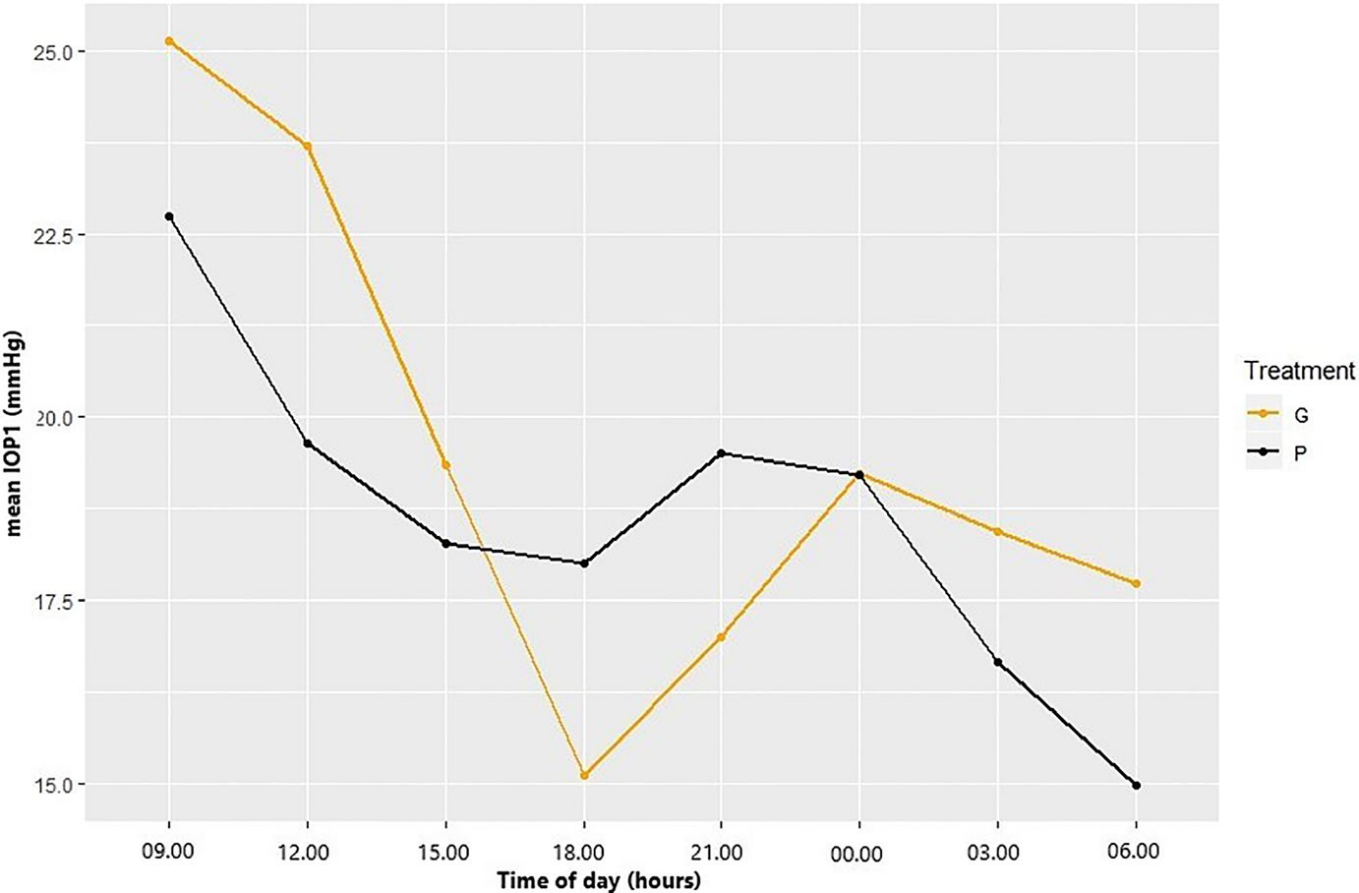
Line diagram depicting mean intraocular pressure (IOP1) in 10 healthy cats at different times of the day for days 1‐5 of the adjustment period (G) and the placebo period (P)

**Figure 2:**
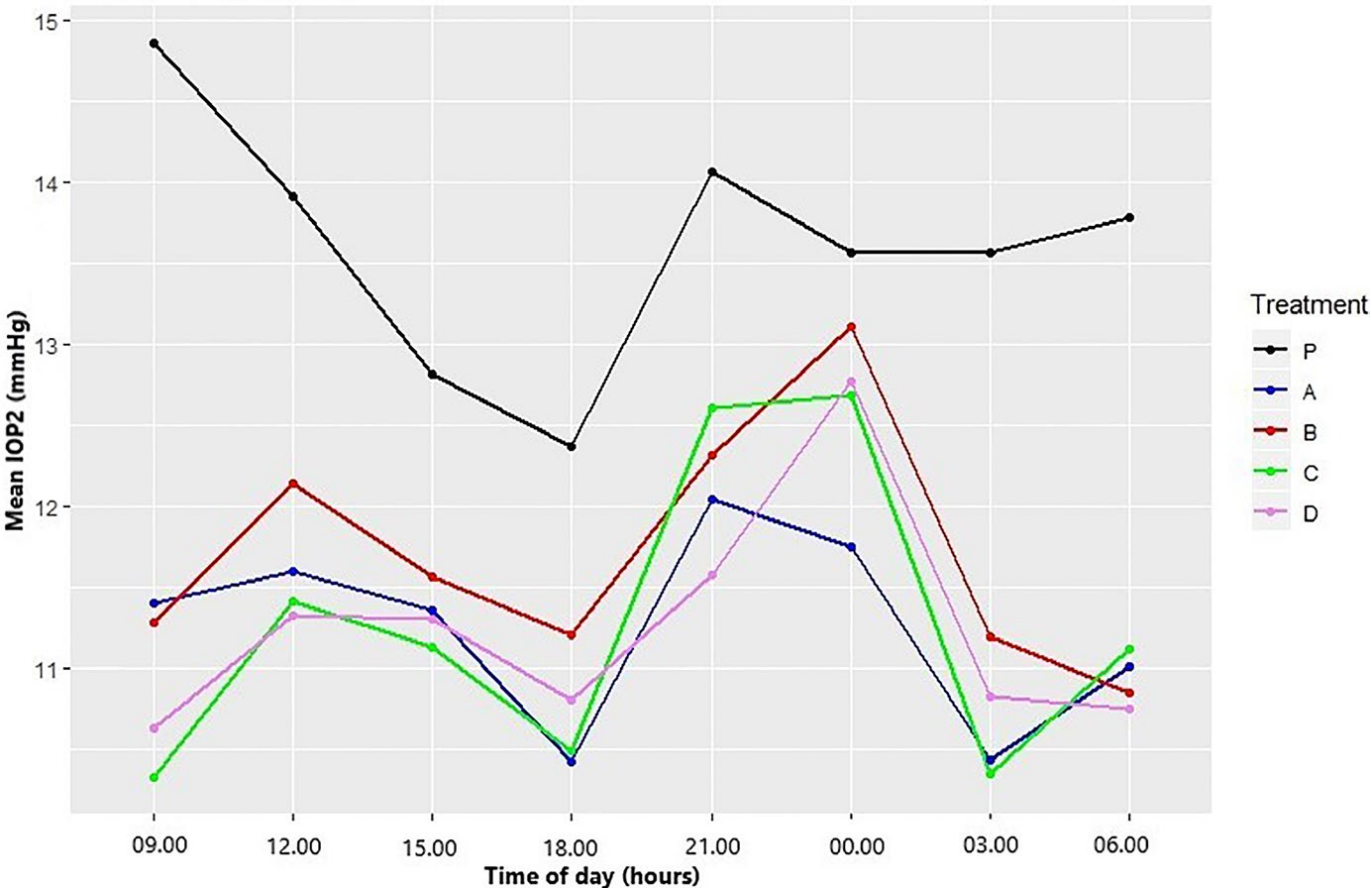
Line diagram depicting mean intraocular pressure (IOP2) in 10 healthy cats at different times of the day, for days 3‐5 of topical treatment with A: dorzolamide 2% q8h; B: brinzolamide 1% q8h; C: dorzolamide 2% q8h and timolol 0.5% q12h; D: brinzolamide 1% q8h and timolol 0.5% q12h; P: placebo (artificial tears)

**Figure 3:**
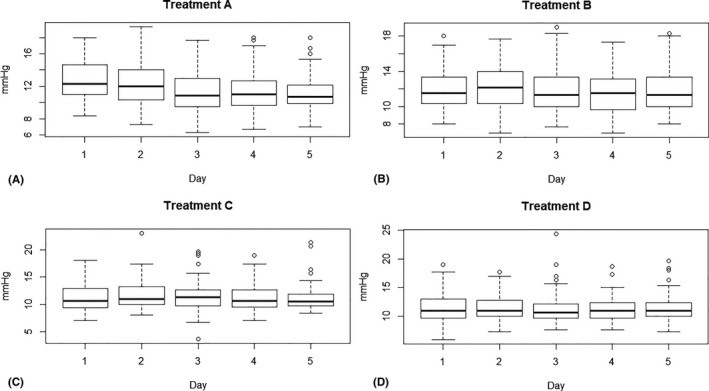
Boxplots depicting mean daily intraocular pressure (IOP2) in 10 healthy cats on days 1‐5 of topical treatment with A: dorzolamide 2% q8h (A); B: brinzolamide 1% q8h (B); C: dorzolamide 2% q8h and timolol 0.5% q12h (C); and D: brinzolamide 1% q8h and timolol 0.5% q12h (D)

Mean baseline days 3‐5 IOP2 of the eye to be treated was 13.6 ± 2.7 mm Hg. All cats demonstrated a circadian rhythm in intraocular pressure during the adjustment and the placebo period (Figure 1). During days 1‐5, IOP1 was highest at 9.00 and lowest at 18.00 for both the adjustment and the placebo period. All treatment protocols resulted in a statistically significant decrease in mean days 3‐5 IOP of the treated eye (Figure 2): A: −2.33 mm Hg (95% CI: −2.71, −1.94), B: −1.91 mm Hg (95% CI: −2.30, −1.53), C: −2.36 mm Hg (95% CI: −2.74, −1.97), and D: −2.37 mm Hg (95% CI: −2.76, −1.98). For all treatment protocols, there was an effect of time of day at three time points (Table 1 and Figure 2B): At time points 21.00 and 00.00, the mean days 1‐5 IOP2 was significantly higher, and at time point 18.00, the mean days 1‐5 IOP2 was significantly lower than at 9.00. Although all treatments clearly lowered the IOP peak value in the morning at 9.00, a significant variation in IOP could still be appreciated during the day (Figure 2). On the first two days of treatment with dorzolamide alone, mean IOP2 decreased to then plateau on day 3. This gradual decrease during the first two to three days could not be appreciated in treatments B‐D (Figure 3). The decrease in IOP following brinzolamide alone seemed slightly smaller than following the other treatment protocols, but the difference with dorzolamide alone was not statistically significant (CI 95%: 0.98‐1.03). The addition of timolol to brinzolamide (treatment D) and dorzolamide (treatment C) resulted in an additional, statistically significant decrease in mean days 1‐5 IOP2 of 5% (CI 95%: 0.93‐0.98) and 4% (CI 95%: 0.94‐0.98), respectively. Maximum IOP‐lowering effect of dorzolamide was reached sooner with added timolol (*P* < 0.01) (Figure 3).

**Table 1:** Results of a linear mixed‐effects regression model that compared the mean IOP2 at different times of the day during days 3‐5 of treatment with topical IOP‐lowering medication (A: dorzolamide 2% q8h; B: brinzolamide 1% q8h; C: dorzolamide 2% q8h and timolol 0.5% q12h; and D: brinzolamide 1% q8h and timolol 0.5%) with the mean IOP2 in the placebo‐treated eye in 10 healthy cats

Variable	Estimate	95% confidence limits	
Intercept (placebo, time 9.00)	13.49	12.76	14.21
Time 12.00	0.38	−0.11	0.87
Time 15.00	−0.06	−0.56	0.44
Time 18.00	−0.64	−1.16	−0.12*
Time 21.00	0.84	0.30	1.38*
Time 00.00	1.10	0.53	1.66*
Time 03.00	−0.41	−1.01	0.19
Time 06.00	−0.18	−0.82	0.46
Treatment A	−2.32	−2.71	−1.94*
Treatment B	−1.91	−2.30	−1.53*
Treatment C	−2.36	−2.74	−1.97*

* = statistically significant

The IOP2 of all cats returned to baseline IOP before initiation of a second, third, or fourth treatment period. Mean IOP2 before treatment A‐D (zero measurement) did not differ significantly from mean IOP2 during the placebo period (*P* = 0.12).

A statistically significant decrease in mean days 3‐5 IOP2 could also be appreciated in the contralateral, nontreated eye, with a mean decrease of −0.19 mm Hg (95% CI: −0.28, −0.11) for dorzolamide alone, −0.18 mm Hg (95%CI: −0.27, −0.10) for brinzolamide alone, −0.31 mm Hg (95%CI: −0.40, −0.23) for dorzolamide and timolol, and −0.24 mm Hg (95%CI: −0.32, −0.15) for brinzolamide and timolol.

### 3.3 Horizontal pupil diameter

Mean pupil diameter during the placebo period from days 3‐5 was 5.9 mm (95%CI: 5.02‐6.84). Timolol induced miosis. Pupil diameter decreased by 9% (CI 95% 0.81‐1.02) when timolol was added to brinzolamide and by a statistically significant 17% when timolol was added to dorzolamide (95%CI: 0.74‐0.93). The interaction effects of light intensity on horizontal pupil diameter were corrected in the analysis.

### 3.4 Heart rate

Mean heart rate measured during the placebo period from days 3‐5 was 156 bpm (CI 95%: 150.30‐169.97). Timolol resulted in a statistically significant decrease in mean heart rate of 8% when added to dorzolamide (CI 95%: 0.87‐0.96) and 5% when added to brinzolamide (CI 95%: 0.90‐1.00).

### 3.5 Ocular irritation

None of the treatment protocols resulted in any signs of ocular irritation.

## 4 DISCUSSION

In this study, we found that all treatment protocols (dorzolamide q8h, brinzolamide q8h, and both carbonic anhydrase inhibitors with added timolol q12h) resulted in a statistically significant decrease in IOP in the treated eye as well as the nontreated eye in healthy cats. Added timolol resulted in an additional small, but statistically significant decrease in IOP.

During the first two days of the adjustment period and the placebo period, IOP readings of all cats were significantly higher than on days 3‐5. Also, the first set of IOP readings (IOP1) was consistently and significantly higher than the second set of IOP readings (IOP2) in all cats. We attributed these findings to the influence of arousal, resulting from handling and measurements in the adjustment period, the instillation of eye drops in the placebo period, and transportation to the examination room at the beginning of each measurement session. In humans, it is well recognized that IOP regulation is under control of the autonomous nervous system,^22^, ^23^ and this was more recently illustrated by the finding of an elevated IOP in university students experiencing examination stress.^24^ As far as the authors know, such effects have not yet been clearly documented in cats.

A circadian rhythm in the IOP of cats, as documented in our study, has been described in previous publications as well,^10^, ^11^, ^14^, ^15^, ^17^, ^18^ although peak values have been observed at different times of day: in the evening,^14^, ^15^, ^18^ during the night,^10^, ^17^ and, in agreement with our findings, in the morning.^11^, ^13^ In other studies, cats were adjusted to a 12‐hour light/12‐hour dark cycle,^10^, ^11^, ^14^, ^17^ and in most, but not all, studies, nighttime measurements were performed under dim red light illumination.^10^, ^14^, ^17^ Environmental light is known to be responsible for various circadian rhythms. In laboratory rabbits, intermittent light exposure at night has been documented to block the endogenous, nightly IOP elevation.^25^ If cats respond similarly, the light exposure during measurements in the dark phase might have influenced the IOP readings in the cats of our study, and possibly those in another study as well.^11^ On the other hand, Del Sole et al^17^ observed that daily variations in the IOP of cats persisted when the cats were kept in constant darkness, suggesting an endogenous clock‐controlled IOP regulation. More research is needed to elucidate the influence of light, or the associated pupil diameter, on IOP in cats.

In addition, the cats’ activity pattern may have influenced the circadian IOP rhythm. The cats in our study were fed at 8.00 and were most active around this time of day. The higher adrenergic tone may possibly have resulted in higher IOP readings at 9.00. In other publications, feeding times and daily activity pattern of the cats were not specified. Further studies are necessary to evaluate different exogenously and endogenously controlled mechanisms that influence IOP.

Dorzolamide and brinzolamide are topically applied carbonic anhydrase inhibitors. They lower the IOP by reducing the formation of aqueous humor in the ciliary body.^8^ Timolol is a topical nonselective β‐adrenergic antagonist, which lowers the IOP, presumably by altering the adrenergic neuronal control of aqueous humor formation by blockade of the β‐receptors in the ciliary body processes.^9^ All three drugs are commonly used for treatment of glaucoma in veterinary patients. Dorzolamide 2% applied q8h resulted in a significant decrease in IOP in the healthy cats of our study. This confirms the findings of previous studies.^10^, ^11^, ^12^ In contrast to other studies,^11^, ^14^ however, we found that brinzolamide 1% applied q8h resulted in a significant decrease in IOP in healthy cats and that the addition of timolol 0.5% q12h to dorzolamide 2% q8h significantly decreased IOP in comparison with topical dorzolamide alone. Our findings partly differ from those of others regarding cats; however, they are in agreement with previous studies in humans and glaucomatous Beagles.^26^, ^27^, ^28^ The reason for the difference in IOP reduction following topical administration of dorzolamide versus brinzolamide in cats is currently unknown, but may possibly be related to differences in their inhibition activity against feline carbonic anhydrase isoenzymes. Further pharmacological research is required.

Combined treatment of brinzolamide 1% q8h and timolol 0.5% q12h resulted in an additional significant decrease in IOP in comparison with brinzolamide 1% q8h alone. As far as the authors know, this is the first study to document this in cats.

In cats with primary congenital glaucoma, diurnal IOP fluctuations are of significantly greater magnitude than in healthy cats.^14^ It is therefore important that topical glaucoma medication reduces these diurnal IOP fluctuations, notably the IOP peaks. In our study, all treatment protocols resulted in a significant decrease in IOP in healthy cats and all treatment protocols were most sufficient in decreasing the peak IOP in the morning. However, a diurnal variation could still be appreciated. Further studies investigating the effects of the topical drugs used in this study on the circadian rhythm in IOP in glaucomatous cats are, therefore, warranted.

Topical administration of timolol 0.5% q24h has been reported to result in significant miosis in healthy as well as glaucomatous cats.^15^, ^16^ The cats in our study also demonstrated a decrease in horizontal pupil diameter if timolol 0.5% was applied q12h in addition to either dorzolamide 2% or brinzolamide 1% q8h, although in the latter combination the difference in pupil diameter was not statistically significant.

Topical application of 0.5% timolol eye drops to healthy cats can cause a decrease in heart rate.^29^ Although clinically significant adverse systemic effects are considered unlikely, monitoring of the heart rate is advised.^29^ In our study, the addition of timolol 0.5% q12h to either dorzolamide 2% or brinzolamide 1% q8h for 5 days resulted in a statistically significant decrease in heart rate, indicating a systemic absorption of the topically applied timolol. The small decrease in mean heart rate did not cause any clinical signs. These results are in agreement with the study by Gunther‐Harrington et al^29^ Another recent study, however, reported no significant reduction in heart rate in normal and glaucomatous cats receiving topical timolol maleate 0.5% gel‐forming solution unilaterally SID for 8 days.^16^ This could either indicate that more frequent topical application of timolol may result in a more marked systemic effect or that aqueous and gel formulations differ in distribution and absorption. This systemic side effect, however, warrants careful use of topical β‐blockers in small animals and animals with known cardiac and/or bronchoconstrictive disease.

One of our male cats was most likely affected with either hypertrophic cardiomyopathy or mitral valve dysplasia. The cat did not receive any systemic medication but remained asymptomatic throughout the study. Reportedly, heart rate in unmedicated cats with asymptomatic hypertrophic cardiomyopathy is not significantly different from heart rate in cats without cardiac abnormalities.^30^ We assumed that intraocular pressure would not be affected, either, but to the best of our knowledge, there are no studies documenting the intraocular pressure in cats with asymptomatic hypertrophic cardiomyopathy or mitral valve dysplasia to support this assumption.

All treatment protocols did not only result in a significant decrease in IOP in the treated eye, but also in a statistically significant decrease in IOP of the contralateral, nontreated eye. A similar finding was reported by Wilkie and Latimer, who observed that the administration of one drop of timolol maleate to one eye resulted in a significant IOP reduction in both the treated and the control eyes of normal cats.^15^ This presumed systemic effect could not be reproduced in a study by Kiland et al^16^ Treatment frequency and/or difference in topical formulation might explain these differences. Although statistically significant, the decrease in IOP of the nontreated eye in the cats of our study was very small. We do not expect this to be of clinical relevance.

Limitations to this study include the small group of cats, the use of nonglaucomatous cats, different levels of experience of the examiners, the use of one TonoVet probe for more than one measurement, practical limitations to the blinding of the examiners, and miosis persisting beyond the washout period.

Following power analysis, the project was started with 12 cats. Due to incompliance with topical medication of two cats, only 10 cats remained available for the study. Nevertheless, the study design and comprehensive dataset allowed for statistically significant results to be reached.

Healthy cats were used, whereas glaucomatous individuals may respond very differently to a certain drug than healthy individuals. From previous studies in normotensive and glaucomatous Beagles and cats, it is known that subjects with glaucoma have an increased sensitivity to IOP‐lowering drugs.^14^, ^31^, ^32^ This suggests that the effects found in the healthy cats of the underlying study may be of a greater magnitude in cats with glaucoma.

At the onset of the study, four of the main examiners had little experience in performing IOP measurements with a TonoVet tonometer. Considering the publication by Görig et al, who reported that TonoVet readings performed by an experienced examiner correlated well with, and were not significantly different from, those performed by an inexperienced examiner, we expected the inter‐examiner variability in our study to be low.^33^ Nevertheless, a standardized protocol was used in order to minimize any differences in animal handling or measurement technique.

Due to financial constraints, one TonoVet probe was used for six consecutive IOP measurements (ie, in two different cats) and then replaced. The probe was not cleaned between measurements in order to prevent inaccurate readings. Since the cats were group‐housed and none of the cats showed any signs of infectious ocular disease, the risk of cross‐contamination with an infectious agent was deemed very small. Taking the brief precorneal retention of topical solutions and the small probe size into account, the probability of transferring medication from one eye to another or from one individual to another was regarded very low as well.^34^ Our findings that, in the Latin square‐based experimental setting, different medication protocols resulted in different effects, and that timolol did not induce miosis in nontreated eyes, support the latter assessment.

The examiners were blinded to the medication they administered, and when performing measurements, they were blinded to the eye receiving the medication. However, blinding may not have been fully effective. Although the original labels of all eye drops were replaced by blank labels, which only mentioned the name of the cat and the eye to be treated, a clear visual distinction could be made between the placebo treatment and the different topical IOP‐lowering drugs as they differed in consistency. Furthermore, since timolol administration resulted in miosis, the examiners were able to point out the timolol‐treated eye. Although a bias cannot be excluded, we do not expect that measurements were affected.

In between treatment protocols, a 3 days’ washout period was maintained. The length of the washout period was based on the few documented observations on late effects of topical carbonic anhydrase inhibitors and timolol, that is, that intraocular pressure and pupil diameter in treated eyes were not significantly different from control eyes 12 hours after a single topical application of a timolol maleate gel‐forming solution 16 and that IOP in treated eyes had nearly returned to the level of untreated eyes within two days after cessation of TID dorzolamide hydrochloride 2% administration, either alone or combined with timolol maleate 0.5% BID.^11^ The washout period proved sufficiently long for the IOP, but not for pupil size to return to baseline values. Although miosis gradually decreased, a slight anisocoria persisted for a week after discontinuation of timolol. As far as the authors know, the sole effect of pupil diameter on IOP is presently unknown. Hence, we cannot be certain that the persistent miosis did not alter our results. However, considering that in all cats IOP returned to baseline values within 3 days after discontinuation of timolol, this does not seem likely.

In conclusion, the healthy cats of our study exhibited a circadian rhythm in IOP with peak values at 9.00. Dorzolamide 2% q8h and brinzolamide 1% q8h, administered either alone or with added timolol 0.5% q12h, resulted in a statistically significant decrease in the IOP of the treated eyes and a statistically significant, but clinically irrelevant decrease in the IOP of the contralateral, untreated eyes. Added timolol resulted in an additional, modest but statistically significant decrease in IOP. The combined administration of dorzolamide and timolol led to a more rapid IOP reduction than the administration of dorzolamide alone, which may be beneficial in cats with glaucoma. Apart from IOP reduction, timolol also caused a decrease in mean heart rate and miosis of the treated eye. Systemic absorption of topical medications is likely, and possible systemic side effects should be taken into consideration when treating a patient.

Our finding that a second set of three IOP readings consistently yielded significantly lower values than a first set of three IOP readings may have implications for both clinical and experimental settings. Stress of any kind likely has a significant effect on IOP in healthy cats and presumably in glaucomatous cats as well. When designing a study in which IOP measurements are involved, or when measuring the IOP in a clinical patient, this effect of stress has to be taken into consideration.
